# Exposure Risks and Preventive Strategies Considered in Dental Care Settings to Combat Coronavirus Disease (COVID-19)

**DOI:** 10.1177/1937586720950746

**Published:** 2020-08-30

**Authors:** Sanjeev B. Khanagar, Ali Al-Ehaideb, Satish Vishwanathaiah, Prabhadevi C. Maganur, Sachin Naik, Salman Siddeeqh

**Affiliations:** 1 Preventive Dental Science Department, College of Dentistry, King Saud Bin Abdulaziz University for Health Sciences, Riyadh, Saudi Arabia; 2 King Abdullah International Medical Research Center, Riyadh, Saudi Arabia; 3 Dental Services, King Abdulaziz Medical City-Ministry of National Guard Health Affairs, Riyadh, Saudi Arabia; 4 Department of Preventive Dental Sciences, Division of Pedodontics, College of Dentistry, Jazan University, Jazan Saudi Arabia; 5 Dental Biomaterials Research Chair, Dental Health Department, College of Applied Medical Sciences, King Saud University, Riyadh, Saudi Arabia; 6 Maxillofacial Surgery and Diagnostic Science Department, College of Dentistry, King Saud Bin Abdulaziz University for Health Sciences, Riyadh, Saudi Arabia

**Keywords:** exposure risks, risk factors, cross contamination, preventive strategies, disease outbreak, dental care settings and coronavirus disease

## Abstract

In recent times, numerous scientific articles have been published on the risks of exposure to infectious microorganisms in dental care settings. The main mode of transmission of such infectious organisms is primarily through bioaerosols generated during routine dental procedures which put both dental care providers and their patients at an increased risk of exposure. Other frequent modes of infection transmission often reported include cross contamination and inadequate adoption of infection control protocols. The main objective of this article is to highlight the findings of those studies that have reported on the routes and modes of transmission of infectious organisms in dental settings, to report possibilities of cross contamination in dental care settings, and also to report any breach in adherence to infection control protocols in dental care settings. We also intend to emphasize on standard infection control protocols and strategies that need to be considered in dental care settings during disease outbreaks like coronavirus disease (COVID-19).

The world has witnessed worst disease outbreaks, but in the modern era, the outbreak of coronavirus disease (COVID-19) is totally devastating and deadly, proving to be a challenge for researchers and healthcare system. The case fatality rates and the contagious nature of this disease have shaken up healthcare facilities, causing economic and social fallouts at the global level.

The outbreak of coronavirus disease (COVID-19) was first reported in Wuhan city, China, in December 2019 (Zhu et al., 2020). The coronavirus (CoV) belongs to the family of Coronaviridae virus and is zoonotic, usually causing a variety of diseases in animals (Fehr & Perlman, 2015; Perlman & Netland, 2009). In humans, it causes upper respiratory tract infections where the infected person presents with symptoms like fever, dry cough, running nose, fatigue, difficulty in breathing, and shortness of breath, and in worst cases it can even result in respiratory arrest and death (Huang et al., 2020).

Human transmission (person to person) primarily through respiratory droplets of the infected person is the major route by which widespread transmission of coronavirus disease (COVID-19) is possible (Jin et al., 2020). The transmission routes of this disease are of major concern to all first-line healthcare providers. There have been four possible routes of transmission of SARS-CoV-2, which mainly includes the direct transmission from the SARS-CoV-2 infected patient with symptoms (symptomatic transmission), direct transmission from the SARS-CoV-2 infected patient without symptoms (presymptomatic transmission), direct transmission from SARS-CoV-2 infected patient who never develops symptoms (asymptomatic transmission), and finally indirect transmission which is not traceable to an index patient (environmental transmission; Ferretti et al., 2020). The transmission of this infectious virus is also through the aerosols along with the droplets, which are exhaled by the infected patients (Leung et al., 2020; Wax & Christian, 2020). Pathogens containing particles/droplets of saliva and mucus are generated when an infected person breathes, coughs, or sneezes. Particles/droplets settle either on surfaces or remain in the air for longer periods, which are inhaled and captured in the respiratory tract leading to infection/occurrence of infectious disease (McCluskey et al., 1996). These droplets may transmit infectious diseases either via indirect contact or droplet transmission in an indoor environment (Chen & Zhao, 2010). Factors such as ventilation rate, airflow pattern, initial exhaled velocity, droplet evaporation, particle component, and indoor droplet dispersion are the key issues to be considered when an individual exposed to infectious droplets (Chen & Zhao, 2010). The authors also suggested that the distribution of all droplets between 0.1 and 200 µm is primarily influenced by ventilation patterns and initial velocity of the droplet, rather than gravity. So, transmission routes of this disease are of major concern to all first-line healthcare providers. Dental care providers are comparatively at a higher risk of exposure to these viruses. This is mainly because the spread of infectious microorganisms in a dental setting could be through bioaerosols generated during dental procedures due to ultrasonic instruments. Other major routes through which infectious microorganisms could spread include direct contact with saliva droplets from infected patients, direct contact with the contaminated instruments or contaminated dental water supply system, and also due to cross contaminations from inanimate surfaces within the dental settings (Peng et al., 2020; Upendran & Geiger, 2020).

***Dental care providers are comparatively at a higher risk of exposure to these viruses. This is mainly because the spread of infectious microorganisms in a dental setting could be through bioaerosols generated during dental procedures due to ultrasonic instruments***

## How Safe Are Dental Care Settings During Disease Outbreaks?

Previous studies reported that severe acute respiratory syndrome coronavirus (SARS-CoV), Middle East respiratory syndrome coronavirus (MERS-CoV), and many other types of influenza viruses have the potential to survive on dry surfaces in healthcare settings for a sufficient time and prevail as potent sources for transmitting infection. The absence of an optimal ventilation system in dental care settings could also aggravate the transmission of these airborne infections (Chan et al., 2011; Van Doremalen et al., 2013).

It is very much essential to consider the pathways of contamination because it could be bidirectional. An infectious microorganism can be transferred from an infected person to a dental care provider or vice versa too, that is, from dental care setting/personal to the patient. Several studies have reported that dental care settings have high cross-contamination potential (Depaola & Grant, 2019; Upendran & Geiger, 2020). The cross contamination is mainly because of the usage of high-speed motor-driven handpieces, ultrasonic instruments, water air sprays, and air polishers that are used in routine dental procedures having a higher potential to generate occupational bioaerosols (Harrel & Molinari, 2004; Rautemaa et al., 2006). These aerosols and droplets from the infected patients have the potential to contaminate the entire surface in a dental care setting. Persistence of CoVs on different types of inanimate surfaces is well reported, which includes dental chair, hand rests, tables, drawers, plastic and wooden surfaces, switches, light handles, latex, cotton, disposables, tissues, gloves, disposable gowns, steel objects, dental instruments, and so on (Otter et al., 2016; Peng et al., 2020).

***It is very much essential to consider the pathways of contamination because it could be bidirectional. An infectious microorganism can be transferred from an infected person to a dental care provider or vice versa too, that is, from dental care setting/personal to the patient.***

## Reports on Contaminated Instruments and Surfaces in Dental Care Settings

The aerators, ultrasonic scaler, and air/water syringes generate potentially harmful bioaerosols during dental procedures that can be a source of exposure to infectious microorganisms. There have been numerous studies reporting on the microbial contamination of dental unit waterline (DUWL) which can potentially act as a source of infection in dental care settings (Table 1).

**Table 1.** Details of the Studies Reporting Contaminated Instruments and Surfaces in Dental Care Settings.

Authors	Year	Instrument/Surface	Number of Specimens Assessed	Number of Specimens Contaminated	Culturing Method	Study Design	Isolates Included	Colony-Forming Unit (CFU) per Component
Sedlata Juraskova et al.	2017	Dental Unit Waterlines (DUWLs)	50 Dental units	18	Not mentioned	Cross-sectional study	Legionella species	Not mentioned
Ajami et al.	2012	DUWLs	52 Dental units	36.1%	Not mentioned	Cross-sectional study	Legionella species	Not mentioned
Smith, G and Smith, A.	2014	Dental handpieces	Not mentioned	Not mentioned	Immersed in phosphate-buffered saline, ultrasonicated, and cultured	Cross-sectional study	Isolates included oral streptococci, pseudomonas species, and *Staphylococcus aureus*	200 CFU per turbine, 400 CFU per spray channel, and 1,000 CFU per item of surgical gear
Herd et al.	2007	Dental handpieces	420	258	Spiral-plated the specimens and incubated them at 37 °C anaerobically and aerobically (with 5% carbon dioxide)	Crossover study	Aerobic bacteria and anaerobic bacteria	Contamination varied from 0 to 6,300 colony-forming units per milliliter

The study by Sedlata Juraskova et al. (2017) estimated the presence of Legionella species in water output of DUWLs at the Institute of Dentistry and Oral Sciences, Faculty of Medicine and Dentistry, Palacky University, Olomouc and University Hospital, Olomouc, and found that 18 of the 50 dental chair units tested positive for Legionella species. Ajami et al. (2012), in his study, estimated the presence of Legionella species in 52 dental units in clinical departments of the Mashhad School of Dentistry and found that 36.1% of dental units were contaminated with Legionella species.

Smith and Smith (2014) stated that the dental handpieces that are used for performing various dental procedures could be an important source of infection transmission if they are not cleaned and sterilized after using them on every patient. In a study, authors assessed microbial contamination of uncleaned dental handpieces and culture report showed colonization of isolates of oral streptococci, pseudomonas species, and *Staphylococcus aureus*. Herd et al. (2007) estimated microbial contamination of low-speed handpiece motors and found that 258 of the 420 specimens produced bacterial growth in culture plates that included aerobic and anaerobic bacteria.

## Reports on Cross Contamination of Infectious Diseases in Dental Care Settings

In a recent report by Meng et al. (2020a), the authors revealed that the School and Hospital of Stomatology, Wuhan University, that provides dental care services confirmed nine coronavirus disease (COVID-19)-infected cases among its employees which included three doctors, three nurses, two administrative staff, and one postgraduate student since this epidemic. The source of infection has not been mentioned. Browning and McCarthy (2012) reported four case reports of cross contamination of herpes simplex virus in a dental care setting. The infection was mainly transmitted from infected patients to dental care providers. In the first case, the dentist complained of herpes whitlow in his arms and hands. Dentist owed to have treated a herpes-infected patient without proper protective wears (gloves). In the second case, the dentist reported suffering from an iatrogenic injury while handling an active case of herpes. He developed herpes whitlow in his arms and hands. In the third case, the dental hygienist was affected by cross contamination from an active case of herpes due to her negligence. The hygienist reported scratching her neck with gloved hand while performing the dental procedure which resulted in whitlow on her neck. In the fourth case, the dental hygienist reported suffering from herpes virus infection in both her eyes. The reason for the transmission was not confirmed in this case.

Roberts et al. (2011) found that 13 of the 65 dental students tested positive for Methicillin-resistant *Staphylococcus aureus* (MRSA) and four of the seven surfaces tested positive for the same. This report suggests that the risk of exposure to these bacteria is higher in dental personnel and their patients in a dental setting as they can act as a reservoir for MRSA.

## Reports on Breach in Adherence to Infection Control Protocols in Dental Care Settings

There are few reports suggesting cross contamination of patients from dental care providers or sometimes dental care settings too. There is a report suggesting transmission of hepatitis B virus within a portable dental clinic in West Virginia where dental care services were provided by licensed dental care providers, dental students, and nonprofessional volunteer staff to roughly around 1,137 patients. The dental services offered were limited to dental cleanings, extractions, and restorations that were done free of cost. After 4 months, a local health department reported several acute hepatitis B virus infection cases among those who had visited a portable dental clinic. The local health department identified five acute hepatitis B virus–infected cases, which included three patients and two volunteers. The investigation revealed numerous breaches in infection control protocols (Radcliffe et al., 2013).

Cleveland et al. (2016) reported numerous transmissions of blood-borne pathogens in dental settings. Here, the authors identified three reports where investigators described the transmission of hepatitis B and C viruses. In both the reports, investigators described single transmission events, that is, from one patient to another in outpatient oral surgery practices. In the third report, investigators described the possible transmission of hepatitis B virus to three patients and two dental care providers in a large temporary dental clinic. The authors identified lapses in infection prevention protocols. Failure to adhere to infection control recommendations by the U.S. Centers for Disease Control and Prevention (CDC) in the dental settings likely led to the transmission of diseases in these identified cases.

A report by Laheij et al. (2012) highlighted a good number of such cases where the authors mentioned that CDC reported attacks of hepatitis B virus infection on 300 patients through hepatitis B virus–infected healthcare workers including dentists and oral surgeons. The authors also mentioned a UK report that documented intraoral and pulmonary tuberculosis cases in patients who had undergone tooth extractions by a dental surgeon suffering from active pulmonary tuberculosis. They also mentioned about a UK report where a dentist carrying multiresistant bacteria posed as a major health risk to patients and coworkers and had also been the potential source for transmission of these bacteria to two patients who had undergone oral surgery procedures. The authors also discussed an outbreak of herpes simplex virus in a dental hygiene setting. A dental hygienist with a herpetic whitlow infected 20 of 46 patients treated, as he did not use gloves during routine practices.

These reports suggest that the risk of exposure to infectious microorganisms in dental care settings is high during disease outbreaks and also suggest that there are higher risks of cross contamination which can affect both dental care providers and patients due to various breaches in infection control protocols.

## Guidelines for Dental Care Providers During Disease Outbreaks Like COVID-19

Doctors are our saviors during pandemics, such as COVID-19. They treat patients risking their lives during many situations (“Infection Control in Health Care Personnel: Executive Summary,” 2019; “Information for Healthcare Professionals About Coronavirus (COVID-19),” 2020; “Improving infection prevention and control at the health facility: Interim practical manual supporting implementation of the WHO guidelines on core components of infection prevention and control programmes,” 2018). But before consulting patients, it is crucial that they must be aware of the guidelines that must be followed during a disease outbreak—this helps to secure their lives and that of patients. In the current scenario, every patient seeking dental treatment should be considered as a potential source of infection since individuals who are not experiencing any symptoms are also reported to transmit the disease. Du et al. (2020) reported that 12.6% of the patients transmitted SARS-CoV-2 virus, even before they showed any symptoms. Considering the infectious nature of the pandemic, stringent infection control protocols have to be adopted in the places that offer dental care services, which include both screening clinics and treatment clinics. Strict infection control protocols have to be adopted in the dental care settings both during and even after outbreaks subsides. Studies have shown that these additional precautionary measures have proven effective in preventing new infections with SARS-CoV-2 virus (Meng et al., 2020a, 2020b).

***Doctors are our saviors during pandemics, such as COVID-19. They treat patients risking their lives during many situations.***

Some important guidelines that must be adhered during a disease outbreak include:

i. Before scheduling an appointment, dental care provider should inquire about the patient’s health condition (presence of cough, cold, fever, etc.).
ii. The patient should also be questioned about their travel history (to any geographical areas affected by COVID-19).
iii. Doctors should encourage patients suspicious of any infections or having recent travel histories to those countries affected by COVID-19 but are asymptomatic and hesitating to get themselves checked.
iv. Dental care providers must adhere to strict and standard infection control protocols.
v. Standard precautions must be taken with all patients.
vi. Every patient must be treated as a potentially infectious candidate.
vii. Dental care providers should adhere to standard personal protective equipment (PPE) to protect their skin and mucous membrane from exposure to infectious organisms and materials. This can be achieved by wearing PPE, such as disposable surgical mouth masks, protective eyewear, face shields, and protective clothing (fluid-resistant disposable gowns), head covering, and shoe coverings.
viii. Should wear surgical masks, eye-protective gears with solid side shields, or opt for complete face shields.
ix. Should change masks in between treating two patients or during patient treatment if it becomes wet.
x. Conventional protective measures may not be sufficient during the respiratory disease outbreaks. Hence dental care providers should have a thorough knowledge of don and doffing of PPE gear, which is a crucial aspect in dental settings. Dental care providers should be trained based on the CDC guidelines for don and doffing of PPE kit, like wearing gowns and gloves covering wrist, using of face shield or respirator, use National Institute for Occupational Safety and Health–approved N95 masks or above, wearing a face shield, and hand hygiene techniques. The CDC also recommends the doffing methods like single-step removal of kit and separate removal, use of glove in glove, or bird beak method for glove removal. Dental care providers can be trained through educational videos, health education pamphlets, and government-initiated training programs (Using Personal Protective Equipment (PPE) CDC., 2019; Hegde, 2020).
xi. Should provide patients with preoperative antimicrobial mouth rinse that can reduce the number of microbes in the oral cavity
xii. Should adhere to strict infection control protocols during patient assessment. The dental personnel should identify patients with acute respiratory illness at check-in. Patients who report with such illnesses should be isolated and made to sit in a well-ventilated room with door fully closed. Disposable surgical masks must be given to persons presenting with signs of influenza or respiratory infections. As a precautionary measure, all patients in waiting areas should be provided with disposable surgical mouth masks and alcohol-based hand sanitizers. The patients should be advised to adhere to respiratory hygiene, cough etiquette, and hand hygiene. Dental care personnel assessing patients with signs/symptoms of respiratory infections should wear standard PPE. Patient and dental personnel should adhere to strict hand hygiene practices using antimicrobial soaps and alcohol-based hand rubs. The dental care providers should strictly practice cleaning and disinfection strategies for environmental management of COVID-19. Dentists must use rubber dams and high-volume saliva ejectors to minimize the spread of aerosols or spatter during dental procedures.
xiii. During dental procedures, negative air pressure minimizes the risk of transmission in healthcare (Cheong & Phua, 2006; Chen & Zhao, 2010). Occupational Safety and Health Administration (OSHA) provides the guidelines for using air filters like HEPA, fiberglass filter, and washable filter; increasing ventilation rate; and to provide negative pressure to minimize the exposure (OSHA guidelines).

## Emergency and Nonemergency Dental Care

Dental care personnel should use their professional expertise and judgment in determining patient’s need for urgent, emergency, or nonemergency care (Solana, 2020).

***Dental care personnel should use their professional expertise and judgment in determining patient’s need for urgent, emergency, or nonemergency care***

### Emergency Dental Care

Dental emergencies are ones that are potentially life-threatening and require immediate dental treatment. Urgent dental care focuses on managing conditions that require immediate attention. This includes uncontrolled bleeding, cellulitis with intraoral or extraoral swelling affecting the patient’s airway, and trauma involving facial bones potentially compromising the patient’s airways. Such critical conditions include severe dental pain from pulpal inflammation, pericoronitis, third molar pain, postoperative osteitis, dry socket, dental abscess resulting in localized pain and swelling, dental trauma (tooth fracture, soft tissue injury, avulsion/luxation), dental treatment prior to critical medical procedures, final crown and bridge cementation (if temporary restorations are lost or broken causing gingival irritations), a biopsy of abnormal tissues, extensive dental caries or defective restorations causing pain, suture removal, denture adjustments (for a patient undergoing radiation therapy/oncology patients when a function is impeded), replacing temporary filling in endodontic access opening in patients experiencing pain, adjusting orthodontic wire/appliances piercing, or ulcerating the oral mucosa.

***Dental emergencies are ones that are potentially life-threatening and require immediate dental treatment***

### Nonemergency Dental Care

Routine or nonemergency procedures include an initial or periodic oral priority to patients needing emergency consultation, and urgent dental care needs that present as life-threatening. Examinations, routine dental cleaning, and preventive therapies, routine orthodontic follow-ups, extraction of asymptomatic teeth, routine dental restorations on asymptomatic carious teeth, and aesthetic dental procedures. These nonemergency procedures should be postponed to avoid the risk of transmission.

***. . . priority to patients needing emergency consultation, and urgent dental care needs that present as life-threatening***

***. . . nonemergency procedures should be postponed to avoid the risk of transmission.***

## Suggestions for Dental Care Providers During Disease Outbreaks

Dental care providers (1) should postpone nonemergency dental care and inform patients about the problems which may arise and (2) should give priority to patients needing emergency consultation and urgent dental care needs that present as life-threatening. (3) Experiencing influenza-like illness (ILI) or with symptoms of respiratory infections should not report to work. Those with ILI should seek medical care by telephone or other remote modes and then report to clinics/hospitals. (4) Experiencing clear signs of the disease should seek medical attention immediately and (5) should follow strict infection control (PPE).

***. . . postpone nonemergency dental care and inform patients about the problems which may arise. . .***

***. . . give priority to patients needing emergency consultation and urgent dental care needs that present as life-threatening***

***Experiencing influenza-like illness (ILI) or with symptoms of respiratory infections should not report to work.***

***Experiencing clear signs of the disease should seek medical attention immediately. . .***

***should follow strict infection control***

## Suggestions for Dental Care Providers Regarding Infection Control Protocols During Disease Outbreaks

### Disinfection in the Patient Receiving Area

To reduce the risk of disease transmission, disinfection of the outpatient areas should be performed every 2 hr using 75% alcohol-based disinfectants or chlorine 500 mg/L or disinfectant wipes effective against SARS-CoV-2 (Zhang & Ling, 2020).

### Cleaning and Disinfection After Dental Care Procedures

#### Dental personnel

After every procedure, dental care providers should remove PPE in sequence according to CDC guidelines and perform hand hygiene throughout the process. Before leaving the dental care setting, it is advised to take a shower or wash hands and face.

#### Clinics/instruments

All dental instruments should be disinfected using standard procedures (disinfection and sterilization). High-frequency contact surfaces such as chairs, tables, computers, sinks, tap, and door handles should be disinfected. This can be done by wiping with a chlorine concentration of 500–1,000 mg/L. Alcohol-containing disinfectants or disposable disinfectant wipes can be used for noncorrosion resistant surfaces. High-frequent contact surfaces should be disinfected for every 2 hr. Dental water lines can be rinsed for 30 s if needed. Air disinfection machine must be turned on or the windows should be kept open for better ventilation during dental procedures. After the working day or lunch hours, clinical area must be disinfected through irradiation using an ultraviolet lamp for 30–60 min, and the windows must be kept open for 30 min. The floor should be disinfected using 500–1,000 mg/L chlorine concentration and kept dry for every 2 hr. Fumigation of the dental clinic should be done periodically. Medical waste management should be followed strictly. The area for storage of medical waste should be cleaned and disinfected using 1,000 mg/L chlorine concentration. Terminal disinfection is the final step at the end of the day of the floor and surfaces of all objects. All the surfaces should be wiped using a chlorine concentration of 1,000 mg/L or disinfectant wipes. In order to disinfect DUWLs, they have to be rinsed for 2 min. Saliva suction pipes, spittoons, and sewage pipes should be disinfected using 500 mg/L chlorine.

## Suggestions for Patients Seeking Dental Care During Disease Outbreaks

***Patients seeking dental care should*** be aware of the risks of exposure to infectious microorganisms during an outbreak of viral diseases. Utilize dental services only for emergency treatments and should postpone any appointments that pertain to regular treatments. Call and inform the dental care provider prior to scheduling an appointment and must ensure that they notify the dentist if they have any symptoms of ILI. Ensure the appointment schedule and visit dental care facility only after confirmation, isolate themselves, and practice social distancing if they have symptoms of disease or confirmed exposure. Seek medical attention immediately if the symptoms persist.

***Utilize dental services only for emergency treatments and should postpone any appointments that pertain to regular treatments.***

***. . . notify the dentist if they have any symptoms of ILI.***

## Conclusion

The purpose of this article is to highlight the risks of exposure to infectious microorganisms during dental procedures and in dental care settings. The risk could be higher during the outbreak of airborne diseases. Both dental care providers and the patients are at an equal risk of exposure to infectious microorganisms due to the bioaerosols generated during dental procedures; they are also at an increased risk of exposure to blood and oral fluids during dental procedures. Hence, dental care providers should adhere to strict infection control protocols and should be extra cautious during disease outbreaks to avoid infectious diseases due to cross contamination. Dental care settings must be maintained hygienically such that they are free from infectious organisms by adopting strict infection control strategies.

***The risk could be higher during the outbreak of airborne diseases. . .***

***. . . dental care providers and the patients are at an equal risk of exposure to infectious microorganisms due to the bioaerosols generated during dental procedures. . .***

***. . . increased risk of exposure to blood and oral fluids during dental procedures***

***Dental care settings must be maintained hygienically such that they are free from infectious organisms by adopting strict infection control strategies.***

## Implications for Practice

- Each patient must be treated as a potentially infectious candidate.
- Only dental emergencies that are potentially life-threatening and require immediate dental treatment should be performed.
- Before planning an arrangement, the dental care provider should enquire about the patient’s well-being condition, and patient receiving department should be informed about screening protocols.
- Furnish patients with a preoperative antimicrobial mouth wash that can lessen the number of microorganisms in the oral cavity.
- The dental care providers should adhere to strict and standard infections control protocols.

*Suggestion*: There should be minimum 30 min of free time between each appointment so that necessary infection protocols can be adopted which can reduce the risk of cross contamination. This enhances safety for dental care providers, assistants, hygienist, and patients.
